# *N*-(4-Fluoro­phen­yl)-2,2-dimethyl­propan­amide

**DOI:** 10.1107/S1600536812020570

**Published:** 2012-05-16

**Authors:** Zheng Fang, Feng Zhang, Bao-hua Zou, Kai Guo

**Affiliations:** a School of Pharmaceutical Sciences, Nanjing University of Technology, Puzhu South Road No. 30 Nanjing, Nanjing 210009, People’s Republic of China; b College of Life Science and Pharmaceutical Engineering, Nanjing University of Technology, Puzhu South Road No. 30 Nanjing, Nanjing 210009, People’s Republic of China

## Abstract

The crystal packing in the title compound, C_11_H_14_FNO, features N—H⋯O hydrogen bonds, resulting in chains of mol­ecules running parallel to the *c* axis. The dihedral angle between the ring and the amide group is 39.1 (3)°.

## Related literature

The title compound is an inter­mediate in the synthesis of ezetimibe, which inhibits the absorption of cholesterol from the intestine, see: Rosenblum *et al.* (1998 ▶). For the synthesis, see: Wang *et al.* (2008 ▶). For a related structure, see: Gowda *et al.* (2007 ▶).
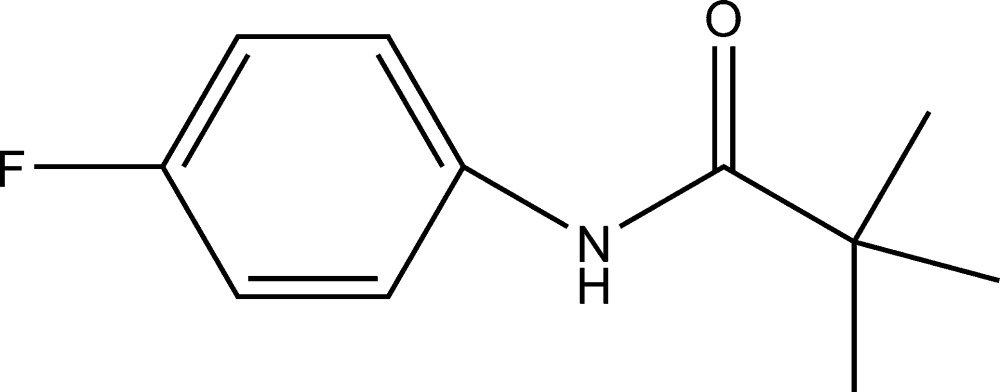


## Experimental

### 

#### Crystal data

C_11_H_14_FNO
*M* *_r_* = 195.23
Monoclinic, 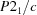

*a* = 9.5750 (19) Å
*b* = 13.027 (3) Å
*c* = 8.8340 (18) Å
β = 92.07 (3)°
*V* = 1101.2 (4) Å^3^
*Z* = 4
Mo *K*α radiation
μ = 0.09 mm^−1^
*T* = 293 K
0.30 × 0.20 × 0.10 mm

#### Data collection

Enraf–Nonius CAD-4 diffractometer
Absorption correction: ψ scan (North *et al.*, 1968 ▶) *T*_min_ = 0.974, *T*_max_ = 0.991
4219 measured reflections
2025 independent reflections
1091 reflections with *I* > 2σ(*I*)
*R*_int_ = 0.082
3 standard reflections every 200 reflections intensity decay: 1%

#### Refinement

*R*[*F* ^2^ > 2σ(*F* ^2^)] = 0.062
*wR*(*F* ^2^) = 0.155
*S* = 1.00
2025 reflections
128 parameters
H-atom parameters constrained
Δρ_max_ = 0.20 e Å^−3^
Δρ_min_ = −0.20 e Å^−3^

### 

Data collection: *CAD-4 Software* (Enraf–Nonius, 1989 ▶); cell refinement: *CAD-4 Software*; data reduction: *XCAD4* (Harms & Wocadlo, 1995 ▶); program(s) used to solve structure: *SHELXS97* (Sheldrick, 2008 ▶); program(s) used to refine structure: *SHELXL97* (Sheldrick, 2008 ▶); molecular graphics: *SHELXTL* (Sheldrick, 2008 ▶); software used to prepare material for publication: *PLATON* (Spek, 2009 ▶).

## Supplementary Material

Crystal structure: contains datablock(s) global, I. DOI: 10.1107/S1600536812020570/pv2534sup1.cif

Structure factors: contains datablock(s) I. DOI: 10.1107/S1600536812020570/pv2534Isup2.hkl

Supplementary material file. DOI: 10.1107/S1600536812020570/pv2534Isup3.cml

Additional supplementary materials:  crystallographic information; 3D view; checkCIF report

## Figures and Tables

**Table 1:** Hydrogen-bond geometry (Å, °)

*D*—H⋯*A*	*D*—H	H⋯*A*	*D*⋯*A*	*D*—H⋯*A*
N—H0*A*⋯O^i^	0.86	2.17	2.990 (3)	159

Symmetry code: (i) 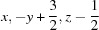.

**Fig. 1.**
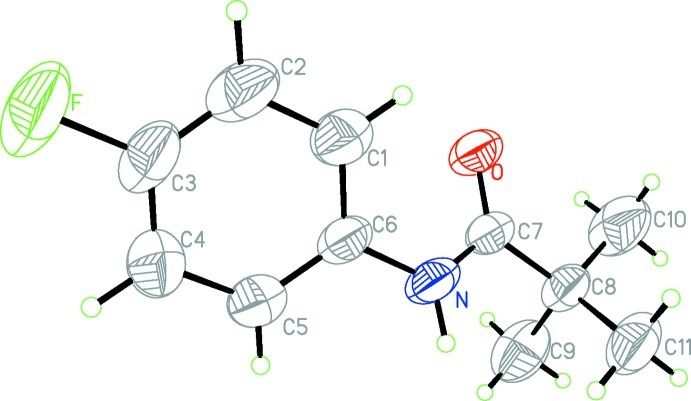
The molecular structure of the title compound with the atom numbering scheme. Displacement ellipsoids are drawn at the 50% probability level. H atoms are presented as small spheres of arbitrary radius.

**Fig. 2.**
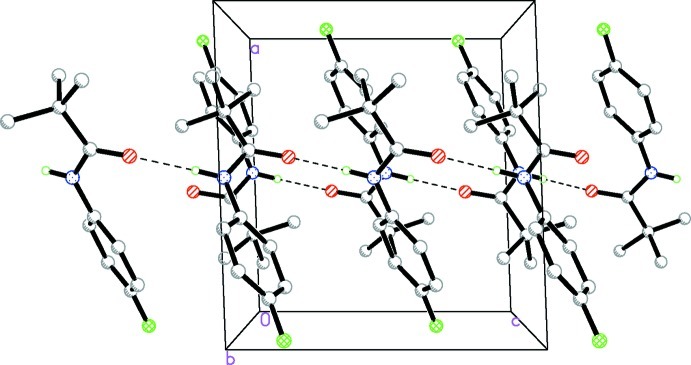
A view of the N—H···O hydrogen bonds (dotted lines) in the crystal structure of the title compound. H atoms non-participating in hydrogen- bonding were omitted for clarity.

## supplementary crystallographic information

### Comment

Ezetimibe is a biologically active molecule and reasearch has shown it to have the useful property of inhibiting the absorption of cholesterol from the intestine (Rosenblum *et al.*, 1998). As a part of our studies on the synthesis of Ezetimibe, the title compound (fIG. 1) which is one of the derivates of an intermediate, has been synthesized and its crystal structure is reported in this paper. The crystal structure of the title compound is stabilized by N—H0A···O hydrogen bonds resulting in chains of molecules running parallel to the *c*-axis (Fig. 1 and Tab. 1). The bond distances and angles in the title molecule are in excellent agreement with the corresponding bond distances and angles reported for its chloro-analogue (Gowda *et al.*, 2007).

### Experimental

To a solution of 4-fluoroaniline (13.32 g, 0.12 mol) in CH_2_Cl_2_(20 ml) were added 4-dimethylaminopyridine (1.2 g, 0.01 mol) and Et_3_N (42.3 ml, 0.31 mol) and cooled the reaction mixture to 273 K. A solution of pivaloyl chloride (14.4 g, 0.12 mol) in CH_2_Cl_2_ (150 ml) was added dropwise over 1 h and the mixture was heated to reflux. After 12 h, H_2_O and H_2_SO_4_ (2 N, 75 ml) were added, separated the layers and washed the organic layer sequentially with NaOH (10%), NaCl (satd.) and water. The organic layer was dried over MgSO_4_ and concentrated to obtain the title compound as a yellow solid product in pure form following the procedure reported earlier (Wang *et al.*, 2008). Crystals of the title compound suitable for X-ray diffraction were obtained by slow evaporation of an ethanol solution.

### Refinement

All H atoms were positioned geometrically and refined using a riding model, with N—H = 0.86 Å and C—H = 0.93 and 0.96 Å, for aryl and methyl H-atoms, respectively. The *U*_iso_(H) were allowed at 1.2*U*_eq_(N/C).

### Crystal data

**Table:**

C_11_H_14_FNO	*F*(000) = 416
*M**_r_* = 195.23	*D*_x_ = 1.178 Mg m^−^^3^
Monoclinic, *P*2_1_/*c*	Mo *K*α radiation, λ = 0.71073 Å
Hall symbol: -P 2ybc	Cell parameters from 25 reflections
*a* = 9.5750 (19) Å	θ = 10–13°
*b* = 13.027 (3) Å	µ = 0.09 mm^−^^1^
*c* = 8.8340 (18) Å	*T* = 293 K
β = 92.07 (3)°	Block, yellow
*V* = 1101.2 (4) Å^3^	0.30 × 0.20 × 0.10 mm
*Z* = 4	

### Data collection

**Table:**

Enraf–Nonius CAD-4 diffractometer	1091 reflections with *I* > 2σ(*I*)
Radiation source: fine-focus sealed tube	*R*_int_ = 0.082
Graphite monochromator	θ_max_ = 25.4°, θ_min_ = 2.1°
ω/2θ scans	*h* = −11→11
Absorption correction: ψ scan (North *et al.*, 1968)	*k* = −15→15
*T*_min_ = 0.974, *T*_max_ = 0.991	*l* = 0→10
4219 measured reflections	3 standard reflections every 200 reflections
2025 independent reflections	intensity decay: 1%

### Refinement

**Table:**

Refinement on *F*^2^	Secondary atom site location: difference Fourier map
Least-squares matrix: full	Hydrogen site location: inferred from neighbouring sites
*R*[*F*^2^ > 2σ(*F*^2^)] = 0.062	H-atom parameters constrained
*wR*(*F*^2^) = 0.155	*w* = 1/[σ^2^(*F*_o_^2^) + (0.065*P*)^2^] where *P* = (*F*_o_^2^ + 2*F*_c_^2^)/3
*S* = 1.00	(Δ/σ)_max_ < 0.001
2025 reflections	Δρ_max_ = 0.20 e Å^−^^3^
128 parameters	Δρ_min_ = −0.20 e Å^−^^3^
0 restraints	Extinction correction: *SHELXL97* (Sheldrick, 2008), Fc^*^=kFc[1+0.001xFc^2^λ^3^/sin(2θ)]^-1/4^
Primary atom site location: structure-invariant direct methods	Extinction coefficient: 0.020 (5)

### Special details

**Table:**

Geometry. All e.s.d.'s (except the e.s.d. in the dihedral angle between two l.s. planes) are estimated using the full covariance matrix. The cell e.s.d.'s are taken into account individually in the estimation of e.s.d.'s in distances, angles and torsion angles; correlations between e.s.d.'s in cell parameters are only used when they are defined by crystal symmetry. An approximate (isotropic) treatment of cell e.s.d.'s is used for estimating e.s.d.'s involving l.s. planes.
Refinement. Refinement of *F*^2^ against ALL reflections. The weighted *R*-factor *wR* and goodness of fit *S* are based on *F*^2^, conventional *R*-factors *R* are based on *F*, with *F* set to zero for negative *F*^2^. The threshold expression of *F*^2^ > σ(*F*^2^) is used only for calculating *R*-factors(gt) *etc*. and is not relevant to the choice of reflections for refinement. *R*-factors based on *F*^2^ are statistically about twice as large as those based on *F*, and *R*- factors based on ALL data will be even larger.

### Fractional atomic coordinates and isotropic or equivalent isotropic displacement parameters (Å^2^)

**Table:**

	*x*	*y*	*z*	*U*_iso_*/*U*_eq_	
O	0.55665 (18)	0.65320 (14)	0.18951 (19)	0.0619 (6)	
F	0.01604 (17)	0.92797 (16)	0.1759 (2)	0.1032 (7)	
N	0.4924 (2)	0.74067 (17)	−0.0211 (2)	0.0588 (7)	
H0A	0.5136	0.7547	−0.1126	0.071*	
C1	0.2795 (3)	0.7359 (2)	0.1236 (3)	0.0638 (8)	
H1A	0.2982	0.6683	0.1514	0.077*	
C2	0.1603 (3)	0.7837 (3)	0.1726 (4)	0.0728 (9)	
H2A	0.0995	0.7493	0.2349	0.087*	
C3	0.1340 (3)	0.8806 (3)	0.1284 (3)	0.0693 (9)	
C4	0.2195 (3)	0.9346 (2)	0.0395 (3)	0.0731 (9)	
H4A	0.1981	1.0016	0.0110	0.088*	
C5	0.3409 (3)	0.8869 (2)	−0.0081 (3)	0.0640 (8)	
H5A	0.4019	0.9225	−0.0687	0.077*	
C6	0.3705 (3)	0.7878 (2)	0.0339 (3)	0.0507 (7)	
C7	0.5775 (3)	0.6763 (2)	0.0568 (3)	0.0496 (7)	
C8	0.7023 (3)	0.6356 (2)	−0.0266 (3)	0.0560 (7)	
C9	0.8029 (3)	0.7255 (3)	−0.0500 (4)	0.0864 (11)	
H9A	0.8316	0.7537	0.0465	0.130*	
H9B	0.7565	0.7775	−0.1102	0.130*	
H9C	0.8834	0.7014	−0.1011	0.130*	
C10	0.7752 (4)	0.5529 (3)	0.0688 (4)	0.1008 (13)	
H10A	0.7121	0.4967	0.0829	0.151*	
H10B	0.8036	0.5809	0.1657	0.151*	
H10C	0.8560	0.5289	0.0181	0.151*	
C11	0.6568 (3)	0.5904 (2)	−0.1808 (3)	0.0759 (10)	
H11A	0.5945	0.5338	−0.1662	0.114*	
H11B	0.7375	0.5669	−0.2321	0.114*	
H11C	0.6097	0.6422	−0.2408	0.114*	

### Atomic displacement parameters (Å^2^)

**Table:**

	*U*^11^	*U*^22^	*U*^33^	*U*^12^	*U*^13^	*U*^23^
O	0.0662 (12)	0.0774 (14)	0.0430 (11)	0.0127 (10)	0.0146 (9)	0.0049 (10)
F	0.0656 (12)	0.1364 (17)	0.1092 (15)	0.0362 (11)	0.0246 (11)	−0.0114 (13)
N	0.0603 (14)	0.0756 (15)	0.0418 (12)	0.0164 (13)	0.0204 (11)	0.0052 (12)
C1	0.0579 (17)	0.071 (2)	0.0637 (18)	0.0048 (15)	0.0160 (15)	0.0065 (15)
C2	0.0509 (17)	0.099 (3)	0.070 (2)	0.0022 (18)	0.0194 (15)	0.0051 (19)
C3	0.0507 (17)	0.095 (2)	0.0631 (18)	0.0197 (18)	0.0119 (15)	−0.0072 (18)
C4	0.073 (2)	0.073 (2)	0.073 (2)	0.0218 (18)	0.0069 (17)	0.0059 (17)
C5	0.0660 (19)	0.071 (2)	0.0564 (17)	0.0073 (16)	0.0177 (14)	0.0078 (15)
C6	0.0519 (15)	0.0640 (18)	0.0370 (13)	0.0053 (14)	0.0113 (12)	−0.0002 (13)
C7	0.0537 (16)	0.0574 (17)	0.0384 (14)	0.0016 (13)	0.0114 (12)	−0.0005 (13)
C8	0.0548 (16)	0.0679 (19)	0.0461 (15)	0.0087 (14)	0.0119 (12)	−0.0050 (14)
C9	0.0548 (18)	0.112 (3)	0.093 (3)	−0.0102 (18)	0.0193 (18)	−0.023 (2)
C10	0.097 (2)	0.125 (3)	0.082 (2)	0.059 (2)	0.022 (2)	0.014 (2)
C11	0.080 (2)	0.082 (2)	0.067 (2)	0.0074 (18)	0.0224 (16)	−0.0181 (17)

### Geometric parameters (Å, º)

**Table:**

O—C7	1.233 (3)	C5—H5A	0.9300
F—C3	1.366 (3)	C7—C8	1.522 (3)
N—C7	1.342 (3)	C8—C10	1.522 (4)
N—C6	1.420 (3)	C8—C11	1.533 (4)
N—H0A	0.8600	C8—C9	1.535 (4)
C1—C6	1.376 (4)	C9—H9A	0.9600
C1—C2	1.383 (4)	C9—H9B	0.9600
C1—H1A	0.9300	C9—H9C	0.9600
C2—C3	1.343 (4)	C10—H10A	0.9600
C2—H2A	0.9300	C10—H10B	0.9600
C3—C4	1.352 (4)	C10—H10C	0.9600
C4—C5	1.397 (4)	C11—H11A	0.9600
C4—H4A	0.9300	C11—H11B	0.9600
C5—C6	1.371 (4)	C11—H11C	0.9600
			
C7—N—C6	125.9 (2)	C7—C8—C10	109.3 (2)
C7—N—H0A	117.1	C7—C8—C11	111.2 (2)
C6—N—H0A	117.1	C10—C8—C11	109.3 (2)
C6—C1—C2	120.4 (3)	C7—C8—C9	107.9 (2)
C6—C1—H1A	119.8	C10—C8—C9	109.7 (3)
C2—C1—H1A	119.8	C11—C8—C9	109.4 (2)
C3—C2—C1	118.7 (3)	C8—C9—H9A	109.5
C3—C2—H2A	120.6	C8—C9—H9B	109.5
C1—C2—H2A	120.6	H9A—C9—H9B	109.5
C2—C3—C4	123.1 (3)	C8—C9—H9C	109.5
C2—C3—F	118.9 (3)	H9A—C9—H9C	109.5
C4—C3—F	117.9 (3)	H9B—C9—H9C	109.5
C3—C4—C5	118.2 (3)	C8—C10—H10A	109.5
C3—C4—H4A	120.9	C8—C10—H10B	109.5
C5—C4—H4A	120.9	H10A—C10—H10B	109.5
C6—C5—C4	120.2 (3)	C8—C10—H10C	109.5
C6—C5—H5A	119.9	H10A—C10—H10C	109.5
C4—C5—H5A	119.9	H10B—C10—H10C	109.5
C5—C6—C1	119.3 (2)	C8—C11—H11A	109.5
C5—C6—N	118.6 (2)	C8—C11—H11B	109.5
C1—C6—N	122.0 (3)	H11A—C11—H11B	109.5
O—C7—N	121.6 (2)	C8—C11—H11C	109.5
O—C7—C8	122.1 (2)	H11A—C11—H11C	109.5
N—C7—C8	116.3 (2)	H11B—C11—H11C	109.5
			
C6—C1—C2—C3	1.4 (5)	C7—N—C6—C5	142.3 (3)
C1—C2—C3—C4	−0.9 (5)	C7—N—C6—C1	−39.9 (4)
C1—C2—C3—F	179.7 (3)	C6—N—C7—O	−1.2 (4)
C2—C3—C4—C5	0.0 (5)	C6—N—C7—C8	−179.9 (2)
F—C3—C4—C5	179.4 (2)	O—C7—C8—C10	9.3 (4)
C3—C4—C5—C6	0.5 (4)	N—C7—C8—C10	−172.0 (3)
C4—C5—C6—C1	0.0 (4)	O—C7—C8—C11	130.1 (3)
C4—C5—C6—N	177.8 (2)	N—C7—C8—C11	−51.3 (3)
C2—C1—C6—C5	−0.9 (4)	O—C7—C8—C9	−110.0 (3)
C2—C1—C6—N	−178.7 (2)	N—C7—C8—C9	68.7 (3)

### Hydrogen-bond geometry (Å, º)

**Table:**

*D*—H···*A*	*D*—H	H···*A*	*D*···*A*	*D*—H···*A*
N—H0*A*···O^i^	0.86	2.17	2.990 (3)	159
C1—H1*A*···O	0.93	2.49	2.904 (3)	107

Symmetry code: (i) *x*, −*y*+3/2, *z*−1/2.
